# The 4.4 Å Capsid Structure of the Giant Melbournevirus Belonging to the *Marseilleviridae* Family

**DOI:** 10.3390/v18040433

**Published:** 2026-04-02

**Authors:** Raymond N. Burton-Smith, Chantal Abergel, Kenta Okamoto, Kazuyoshi Murata

**Affiliations:** 1 Exploratory Research Center on Life and Living Systems (ExCELLS), National Institutes of Natural Sciences, Okazaki 444-8585, Japan; ray@nips.ac.jp; 2 National Institute for Physiological Sciences, National Institutes of Natural Sciences, Okazaki 444-8585, Japan; 3 Department of Physiological Sciences, School of Life Science, The Graduate University for Advanced Studies (SOKENDAI), Okazaki 444-8585, Japan; 4 Structural and Genomic Information Laboratory, UMR 7256 (IMM FR 3479, IM2B, IOM), Centre National de la Recherche Scientifique, Aix-Marseille University, 13288 Marseille, France; chantal.abergel@igs.cnrs-mrs.fr; 5 Program in Molecular Biophysics, Department of Cell and Molecular Biology, Uppsala University, 75123 Uppsala, Sweden; kenta.okamoto@icm.uu.se

**Keywords:** giant virus, NCLDV, capsid proteins, cryo-electron microscopy, single-particle analysis, block-based reconstruction

## Abstract

Members of *Marseilleviridae*, a family of icosahedral giant viruses, have been identified worldwide in all types of environments. The virion shows a characteristic internal membrane extrusion at the five-fold vertices of the capsid, but its structural details need to be elucidated. We now report the 4.4 Å cryo-electron microscopy structure of the melbournevirus capsid by using a block-based reconstruction approach. Results: An atomic model of the major capsid protein (MCP) shows a unique cup structure on the trimer that accommodates additional proteins. A polyalanine model of the Penton base protein shows internally extended N- and C-terminals, which indirectly connect to the internal membrane extrusion. The Marseilleviruses share the same orientational organization of the MCPs as previously reported for other giant viruses, but the unique minor capsid protein components named Scaffold may be alternatively utilized to control the dimensions of the capsid during assembly as the tape measure protein.

## 1. Introduction

Members of the *Marseilleviridae* family belonging to the nucleocytoplasmic large DNA viruses (NCLDVs) [1] infect *Acanthamoeba* [2] and present a highly complex ~360 kbp genome and large icosahedral capsid with a diameter of ~250 nm. The group of NCLDVs initially proposed by Iyer et al. [3] is currently classified into the *Nucleocytoviricota* realm (PMID: 31187277) [4] and suggested to consist of seven families and several subfamilies of viruses [5], which commonly possess double-stranded DNA and target a wide range of eukaryotic hosts [6]. Since the first discovery of Marseillevirus in 2009 [7], the *Marseilleviridae* has expanded to more than 10 members classified into five lineages, A to E [8,9]. Members of *Marseilleviridae* have been found in some human patients, but their link with a particular disease is yet to be identified [10,11,12].

Melbournevirus is a member of the *Marseilleviridae* isolated from a freshwater pond in Melbourne, Australia, in 2014 [13]. It shares common genomic and structural features with the other members of the family and is classified into lineage A. The cryo-electron microscopy (cryo-EM) structure was reported at 26 Å resolution by using a 200 kV microscope [14], revealing the major capsid protein (MCP) array with a triangulation number of the icosahedral capsid as T = 309. In addition, characteristic morphological features of melbournevirus capsids were elucidated, including extrusion of the internal membrane at the five-fold vertices of the capsid and a nucleoid containing a large density body, the nature of which has not yet been elucidated.

The sub-nanometer resolution structure of tokyovirus, one member of the *Marseilleviridae*, has been reported using high voltage cryo-electron microscopy (cryo-HVEM) [15]. The map did not reach a high enough resolution to be able to build de novo the capsid structures consisting of the various minor capsid proteins (mCPs) and only allowed a semi-fitted model for the MCP and a hypothetical fit of the Paramecium Bursaria Chlorella Virus 1 (PBCV-1) Penton protein [15]. Since manual data collection with the cryo-HVEM microscope restricted the number of images collected, the current resolution was limited to ~8 Å, even after using a capsid-focused mask. However, the tokyovirus cryo-EM map elucidated the novel capsid protein network of the giant virus, including the Scaffold protein components, which could assist in the formation of the characteristic internal membrane extrusion at the five-fold vertices of the capsid and function in a similar manner to a tape measure protein [16]. Furthermore, it suggested that a glycosylated cap protein was located on top of the MCP trimer [15].

High-resolution cryo-EM maps of the giant viruses have been reported in three structures exceeding 5 Å resolution: 3.5 Å for PBCV-1 [17], 4.6 Å and 4.8 Å for African swine fever virus (ASFV) [18,19]. These structures utilized 300 kV microscopes and were reconstructed with a technique termed “block-based” reconstruction [20] to improve the obtained resolutions. Leveraging the high symmetry of icosahedral viruses, this technique can reduce the size of the boxes required for 3D reconstruction and enables localized defocus refinement across the particles. For all objects >150 nm, this defocus gradient is considerable and has a negative impact on the attainable resolution [21]. Consequently, they successfully constructed the atomic structures of the MCP and several mCPs in PBCV-1 and ASFV. In PBCV-1, the structures of 13 different types of mCP were identified in addition to the MCP [17], while in ASFV, the structures of four mCPs were determined in addition to the MCP [18,19].

Here, we report the structure of melbournevirus to 4.9 Å for the whole particle, with only 3124 particle images, and a maximum resolution of 4.42 Å obtained on the acquired dataset for the five-fold, three-fold, and two-fold “blocks”, using symmetry expansion, recentering, and defocus refinement. This is functionally equivalent to block-based reconstruction [20], which has been applied to the study of other giant viruses but operates entirely within the RELION processing suite [22,23,24,25]. The capsid, particularly in the block-based reconstructions, permits clearer views of the complex lattice of capsid proteins. While the hard limit of resolution in this data makes residue identification difficult, polyalanine chain models could be generated in some cases. An atomic model of the MCP and a polyalanine model of the Penton base protein, which has no identified polypeptide sequence, were built and compared with other giant viruses reported previously. While the Marseilleviruses share the same orientational organization of the MCPs as PBCV-1 [16] and Cafeteria roenbergensis virus (CroV) [26], the unique minor capsid protein component named Scaffold may be alternatively used to control the dimensions of the capsid during assembly. These structural differences in the capsid demonstrate the variety and biological flexibility of the giant viruses, while simultaneously utilizing similar strategies for some critical features. The cryo-EM map of melbournevirus provides clearer views of the complex lattice of the capsid and the molecular interactions between capsid proteins, revealing the functional viral capsid network.

## 2. Materials and Methods

### 2.1. Viral Propagation and Purification

Melbournevirus particles were propagated and purified as described previously [13,14]. It is described briefly as follows. Melbournevirus particles were propagated in *Acanthamoeba castellanii* cells that were cultured in PYG medium and confluent in ten 75 cm^2^ culture flasks. The infected culture fluid was collected three days post infection and centrifuged for 10 min at 500× *g*, 4 °C. The supernatant was centrifuged for 35 min at 6500× *g*, 4 °C, and the pellet was suspended in 1 mL of PBS (−). The sample was ultracentrifuged in 10–60% (*w*/*v*) continuous sucrose gradient for 90 min at 6500× *g*, 4 °C. The virus particles’ band was collected from the sucrose gradient and centrifuged for 35 min at 6500× *g*, 4 °C. The pellet was rinsed five times in 50 mL of PBS (−) by centrifugation to remove excess sucrose. Finally, the virus particles were suspended in 100 μL PBS (−) for further cryo-EM analysis.

### 2.2. Cryo-EM Sample Preparation and Acquisition

An amount of 3 μL of purified virus particles was loaded on a glow-discharged lacey carbon copper grid and flash-frozen in liquid ethane after 2 s blotting in 100% humidity at 4 °C using a Vitrobot Mark IV (Thermo Fisher Scientific, Waltham, MA, USA). The particles images were collected using a 300 kV Titan Krios G2 microscope (Thermo Fisher Scientific) equipped with a K2 Summit direct electron detector using counting mode and a GIF energy filter of 20 eV slit width (Gatan Inc., Pleasanton, CA, USA). Two datasets were collected, dataset 1 with 20 frames per micrograph movie, and dataset 2 with 40 frames per micrograph movie. These datasets were taken at a nominal magnification of 64,000× (pixel size on specimen is 2.21 Å/pixel), at 0.8–3.0 μm under focus, and at 26.4 e−/Å^2^ total dose.

### 2.3. Image Processing

The image processing workflow of the whole virus particle is summarized graphically in Figure S1. A total of 3367 micrograph movies were imported into RELION 3.1 [24,25], and motion correction was performed using MotionCor2 1.4.0 [27] using whole-frame motion correction. Contrast transfer function (CTF) parameters were calculated on the resulting micrographs using CTFFIND 4.1.14 [28], using the RELION 3.1 default parameters except for defocus step size, which was set to 100 Å. A total of 229 particles were manually picked, extracted using 4× downsampling (to 8.84 Å/pixel in 360-pixel boxes), and 2D-classified into 10 classes with 0.5° angular sampling and “ignore CTFs until first peak” enabled. Three classes were used to generate three initial models using I3 symmetry with a mask diameter of 2550 Å and an initial angular sampling of 3.7°, of which the best was chosen. These three clear classes, comprising 182 particles, were used as references for RELION template autopicking of the full dataset, resulting in 7000 particles picked. These were extracted using 4× downsampling and 2D-classified into 40 classes with a mask diameter of 2650 Å, angular sampling of 1°, and “ignore CTFs until first peak” enabled. A total of 19 classes, totaling 5047 particles, were selected in clear classes and subjected to a further round of 2D classification, this time with 0.5° angular sampling and full CTF correction. This resulted in 3 clear classes, containing 3852 particles. 3D classification into five classes was carried out with an angular sampling of 1.8° and “ignore CTFs until first peak” enabled. Three classes, totaling 3569 particles, were selected from the 3D classification, and a 3D refinement was carried out. The maximum resolution possible with the downsampled particles was achieved (17.68 Å). Particles were re-extracted using 2× downsampling (4.42 Å/pixel in 720-pixel boxes) and a further 3D refinement and single round of per-particle defocus refinement again achieved the maximum resolution possible with downsampled particles (8.84 Å). Particles were re-extracted without downsampling (2.21 Å/pixel), which permitted a reconstruction to achieve 7 Å. Unfortunately, CTF refinement with 1440-pixel boxes would fail. Particles were re-extracted with 1.5× downsampling (3.315 Å/pixel in 960-pixel boxes) and anisotropic magnification distortion estimated, followed by further defocus refinement. This resulted in a 6.65 Å reconstruction. 3D classification with alignment disabled was carried out into five classes, with the two best classes, comprising 3124 particles, selected, resulting in a 6.45 Å reconstruction. Ewald sphere correction was used with *relion_reconstruct* to generate independent half-maps using the parameters from this final refinement, which results in a final gold standard FSC [29] global estimated resolution of 4.9 Å with a soft capsid-only mask.

At this point, we moved toward a strategy akin to that of block-based reconstruction [20], but only using the functions contained within RELION 3.1 [24]. The image processing workflow of the block-based reconstruction is summarized in Figure S2. First, symmetry expansion was carried out. Then, three masks individually focused on five-, three-, and two-fold axes were generated in UCSF Chimera 1.14 [30] before particle subtraction was used to calculate the shifts to center the focused region in the box. Particle subtraction was canceled after shifts were calculated. Using these RELION-calculated shifts, the five-, three-, and two-fold axes were extracted in 440-, 500-, and 700-pixel boxes, respectively, each containing a total of 187,440 boxes. All block reconstructions were carried out with C1 symmetry imposed. The *relion_reconstruct* module was used for each extracted particle set to generate a reference for refinement and check that the positioning was correct. 3D refinements were carried out for each segment, achieving 4.8–5 Å with post-processing. Defocus refinement was carried out once for each, and *relion_reconstruct* was used to reconstruct half-maps using the defocus-refined particle parameters. The five-fold and three-fold reconstructions achieved 4.42 Å, the maximum resolution possible with these data. The two-fold reconstruction achieved 4.43 Å, likely caused by the larger box size used. Further CTF parameter optimization and Bayesian polishing were not carried out.

### 2.4. Model Building

The amino acid sequence of the MCP of melbournevirus is annotated, along with four other candidates which may be mCPs previously reported by Okamoto et al. [14]. A homology model of the MCP was generated using SWISS-MODEL (online at https://swissmodel.expasy.org/, accessed on 31 March 2026) [31] using a structural template of that of PBCV-1 (PDBID: 5TIQ). This model was rigid-body-fit into a SEGGER 2.5.3-extracted [32] MCP from the center of the three-fold axis and fitted into the map using ISOLDE 1,0b5 [33] with restraints on predicted secondary structure. Real-space refinement was carried out in PHENIX 1.17 [34], with intermediate rounds of geometry minimization.

The PDB model of the PBCV-1 Penton protein was rigid-body-fit to the melbournevirus Penton density. It was deemed a poor fit, so the map blocks were segmented using SEGGER 2.5.3 [32], and extracted segments were processed with DeepTracer (online at: https://deeptracer.uw.edu, accessed on 31 March 2026) [35] using a polyalanine sequence for chain tracing. The best of these polyalanine chain models was rigid-body-fit to fill the Penton density with five identical chains. ISOLDE 1.0b5 [33] was used to better fit some sections of the chain. Real-space refinement and validation were carried out in PHENIX 1.17 [34]. Other parts of the mCPs (PC-α and the Lattice protein) were also processed in the same way and the polyalanine models were built.

## 3. Results

### 3.1. The Structure of Melbournevirus Capsid

**Figure 1:**
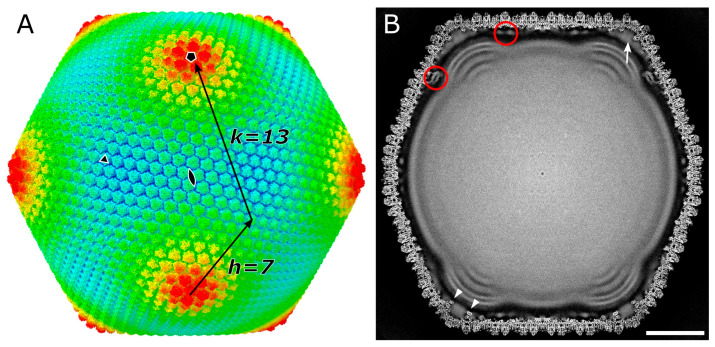
SPA 3D reconstruction of melbournevirus at 4.9 Å. (**A**) External view of melbournevirus, colored by radius with the following parameters: blue, 1050 Å; turquoise, 1100 Å; green, 1150 Å; yellow, 1200 Å; red, 1250 Å. Symmetry axes are marked by a pentagon (five-fold), triangle (three-fold), and double teardrop (two-fold). The indices of h and k for the triangulation number are indicated. (**B**) Central slice of melbournevirus. Red circles indicate the Scaffold proteins between the capsid layer and inner membrane. A white arrow indicates a low-resolution density, which fills a space between the pentasymmetron and the inner membrane extrusion but does not directly contact the inner membrane. White arrowheads indicate PC-α, one of the mCP components of the pentasymmetron mentioned in those of tokyovirus [15]. Scale bar equals 50 nm.

The image processing flowchart and the dataset collection details of the whole-virion 3D reconstruction are shown in Figure S1 and Table S1. Using Ewald sphere correction, a 4.9 Å global resolution map of the melbournevirus virion was reconstructed by assuming icosahedral symmetry (Figure 1 and Figure S1F). Melbournevirus shares a high structural similarity to that of tokyovirus, another member of the *Marseilleviridae*, reported at 7.7 Å resolution [15], possessing the same MCP pattern with the same triangulation number (T = 309) on the capsid surface. The internal membrane shows the characteristic extrusion at the five-fold axis, where the Scaffold protein network between the capsid layer and the internal membrane is comparable to that of tokyovirus (red circles in Figure 1B). A low-resolution density fills a space between the pentasymmetron and the internal membrane extrusion (arrow in Figure 1B), although it does not directly contact the internal membrane. These components may be relatively flexible in the structure because they are not well resolved in the map.

**Figure 2:**
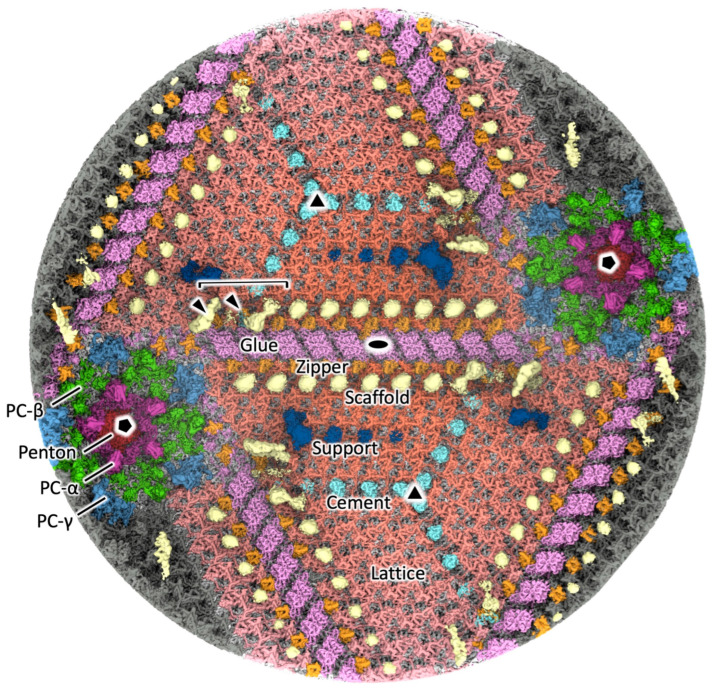
Segmentation of the mCPs in melbournevirus, focused on the asymmetric units encompassing the five-, three-, and two-fold axes, and viewed from the inside of the capsid. The map was filtered by local resolution to permit visualization of the flexible Scaffold and Support proteins, which are otherwise weak due to uniform b-factor sharpening of the map. Support (dark blue), Scaffold (yellow), Zipper (orange), Glue (pink), Cement (cyan), Lattice (salmon), PC-α (red purple), PC-β (green), PC-γ (sky blue), and Penton indicate the names of each mCPs mentioned in those of tokyovirus [15]. Five-fold, three-fold, and two-fold axes are indicated by a black pentagon, triangle, and double teardrop, respectively. The bracket shows the “horseshoe”-shaped head of the Scaffold proteins.

With this melbournevirus dataset, we approached the resolutions at which de novo model building can be attempted. Using the “block-based” reconstruction strategy [20] permitted further improvements in resolution (Figure S2). Blocks were reconstructed with no symmetry applied (C1), with a soft mask focused on the capsid. Carrying out the block reconstructions without defocus refinement barely improved resolution above that of the Ewald-sphere-corrected whole-virus reconstruction, where the global Fourier shell correlation (FSC) [23] for the five-fold block estimated was 4.8 Å. However, a single pass of defocus refinement resulted in reconstructions reaching the maximum resolution of 4.42 Å (Figures S2–S5, Table S2). Figure 2 shows the capsid viewed from the inside, which is merged with the block reconstructions of the two-fold (Figure S3), three-fold (Figure S4), and five-fold (Figure S5) symmetry axes and filtered by local resolution, revealing the details of the protein network formed with mCPs. Each component is labeled according to the nomenclature proposed in the segmentation of tokyovirus by Chihara et al. [15] and closely follows the motifs previously described. These mCPs are roughly classified into two components: ones closely associated with the surface MCP and others located between the surface capsid and internal membrane. The former components consisted of Glue, Zipper, Cement, Lattice, Penton, PC-α, PC-β, and PC-γ, which show higher resolution (Figures S3–S5), exhibiting the clear polypeptide densities (Figure S6). By contrast, the latter components consisted of Scaffold and Support, which show lower resolution (Figures S3–S5), suggesting that these structures are relatively flexible between the rigid surface capsid and the elastic internal membrane (Figure 2). All capsid components are individually detailed as follows.

### 3.2. The Structure of MCP

**Figure 3:**
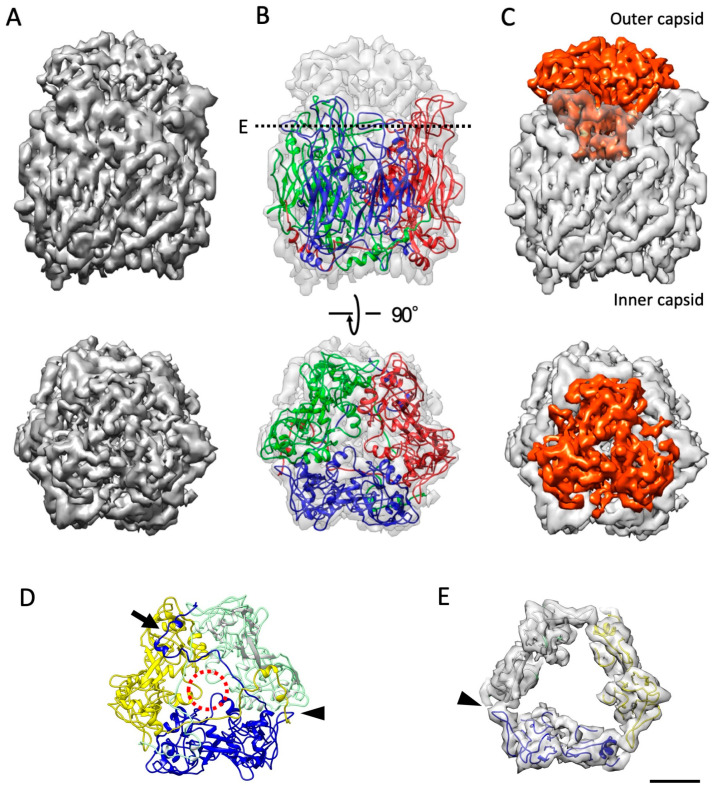
Melbournevirus MCP, with the homology model fitted. (**A**) An MCP trimer extracted from the center of the three-fold axis. (**B**) Transparent density with the homology model fitted. (**C**) The MCP with the “cap” region, where the areas not annotated with the MCP sequence are highlighted in red. Top and bottom panels show the side and top views of the MCP. (**D**) The FG1 loops (dotted red circle), N-terminal domain (arrow), and HI1 loop (arrowhead) stabilize the MCP trimer. The trimer models are viewed from the inner capsid side. (**E**) A sliced map with the models of the MCP viewed at the dotted line in (**B**) and from the outer capsid side. The HI1 loop interacts with the adjacent HI2 loop and forms a cup structure with the extended DE1 and FG1 loops to accommodate the cap density (red in panel (**C**)). Scale bar equals 2 nm.

The amino acid sequence of the melbournevirus MCP was aligned against those of tokyovirus [15], PBCV-1 [17] and other NCLDVs, where it scores highly against tokyovirus, and within acceptable parameters against PBCV-1 and Iridovirus [36] (Figure S7 [37,38]). The melbournevirus MCP was predicted to possess the same “double jelly roll” motif as other NCLDV MCPs [5] (Figure S8). This is confirmed by the striated density on the MCP trimer extracted from the center of the three-fold axis (Figure 3A), which describes a β-sheet structure. A homology model of the melbournevirus MCP was built by using the PBCV-1 MCP (PDB ID: 5TIQ) as a structural template, flexibly fitted to the density, clearly showing the molecular model of the MCP trimer (Figure 3B). As previously reported for PBCV-1 [17] and ASFV [19], the MCP trimer is formed by the interactions between the outermost FG1 loops of MCP (Figures S7 and S8), which extend inside the trimer with two short α-helices (dotted red circle in Figure 3D). The interaction between the MCP trimer is further stabilized with the innermost N-terminal domains (NTDs) of the MCP, which are extended from the adjacent monomers (arrow in Figure 3D).

Furthermore, in the melbournevirus MCP trimer, the elongated HI1 loop interacts with the adjacent MCP monomer (arrowheads in Figure 3D,E and Figure S8A) and forms a unique cup structure with the relatively long loops of DE1 and FG1 on the top of the MCP trimer (Figure 3E). The cup structure serves to accommodate a similarly uniform and symmetric “cap” region to tokyovirus [15], which cannot be attributed to the MCP polypeptide (red in Figure 3B,C). Previously, Okamoto et al. reported that an uncharacterized protein, MEL_236, appeared to have the same abundance as the MCP protein [14]. At 16 kDa, MEL_236 is approximately the correct size (given a margin of error for SDS-PAGE size determination) to correspond with the PAS stain-sensitive 14 kDa protein of tokyovirus previously reported [15]. Genomic studies of noumeavirus reported the ortholog of this, NMV_189, as the most abundant protein [9,39]. Unfortunately, in these maps, the cap region is not clear enough to build a model from this sequence (red in Figure 3C, arrowheads in Figures S3D and S4D).

### 3.3. The Structure of the Penton Base Protein

**Figure 4:**
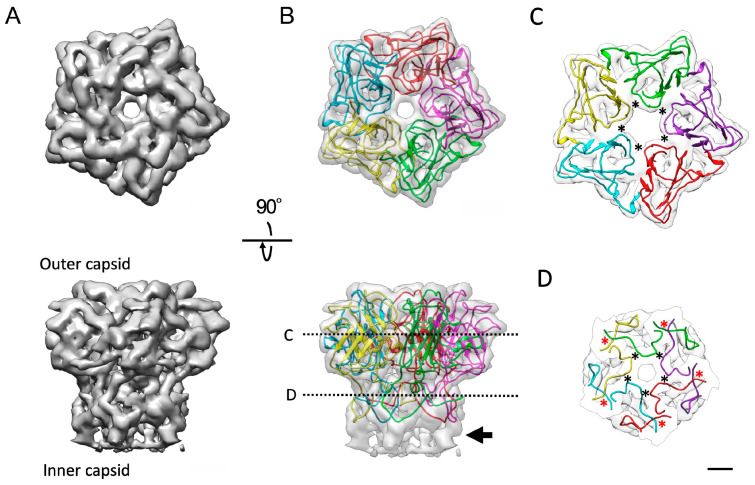
A pentamer of Penton base proteins of melbournevirus. (**A**) The extracted Penton density. (**B**) The map with a de novo polyalanine chain model built into the individual symmetrical densities. Top and bottom panels are viewed from the top and the side of the pentamer, respectively. The arrow indicates that the density does not fill the polyalanine model. (**C**) The loops from the individual Penton base proteins externally stabilize the pentamer. The sliced position is indicated in panel (**B**). The interaction points are labeled with asterisks. (**D**) The interactions between N- and C-terminals internally stabilize the pentamer. The sliced position is indicated in panel (**B**). The interaction points between N-terminals and between N-terminal and C-terminal are labeled with black and red asterisks, respectively. Each individual polyalanine chain is displayed in a different color. Scale bar equals 1 nm.

As reported in other giant virus capsids [15,17,19,36], the five-fold axis of the melbournevirus capsid is filled with a Penton base protein pentamer. We performed a rigid-body fit of the Penton base protein from PBCV-1 [17] to this density, but the quality of the fit was marginal. Therefore, the penton density was extracted from the five-fold block reconstruction (Figure S3) using UCSF Chimera 1.14 [30] and SEGGER 2.5.3 [32], and DeepTracer (online at: https://deeptracer.uw.edu, accessed on 31 March 2026) [35] was used with this extracted density together with a short polyalanine sequence to trace the backbone (Figure 4). The Penton protein exhibited a single jelly roll (SJR) motif [40] similar to those of other giant viruses (Figure S9). The pentamer is formed by two interactions, external and internal; the external is the loop extended inward of the pentamer in the outer capsid region (black asterisks in Figure 4C), and the internal is the interactions between the N-terminals (black asterisks in Figure 4D) and between the N-terminal and the adjacent C-terminal (red asterisks in Figure 4D). The entire structure of the Penton base protein is similar to that of PBCV-1, which does not have the large insertion domain found in CroV-dependent mavirus (Figure S9C), though the homologous protein of the PBCV-1 Penton has not been identified in melbournevirus. In the cryo-EM map, some inner capsid densities also exist under the Penton base protein models (arrow in Figure 4B). Another component may exist in this region connecting the Penton to the internal membrane extrusion.

The five-fold axis of melbournevirus capsid with the characteristic extrusion of the internal membrane shows a low-resolution density filling the area underneath the Penton, completely enveloping the pentasymmetron component proteins (arrow in Figure 1B), but this density does not directly contact the internal membrane extrusion.

### 3.4. The Minor Capsid Proteins Underneath the Pentasymmetron

As the minor capsid proteins underneath the pentasymmetron, three pentasymmetron protein components (PC-α, PC-β, PC-γ) have been identified in tokyovirus [15]. Similar components were also identified in melbournevirus (Figure 2 and Figure S10). With the improved resolution of the melbournevirus block-based reconstruction, we can clearly see one of the pentasymmetron protein components (named PC-α) revealing two quadruple bundles, stacked one on top of the other (PC-α in Figure 2 and Figure S10). A polyalanine model was built de novo and fitted into the density (Figure S6). However, it is less clear whether this is a single protein or two independent ones, as the extremity of this region is relatively unsupported and, as a result, the resolution is ~6 Å (Figure S3). Our current hypothesis is that they are two independent bundles, where the polypeptide chain continues into the capsid framework (Figures S6 and S10), which are located around the internal membrane extrusion (arrowheads in Figure 1B).

### 3.5. Other Minor Capsid Protein Components

Like tokyovirus, the mCPs of melbournevirus form an intricate lattice network (Figure 2 and Figure S10). The two viruses, extremely close phylogenetically, should demonstrate high structural similarity. Indeed, melbournevirus shows the same lattice array, with the trapezoidal lattice consisting of the Lattice protein components (salmon in Figure 2 and salmon pink in Figure S10), which are linked by Cement protein components (pale blue in Figure 2 and Figure S10) and surrounded by Glue/Zipper protein components (pink and orange in Figure 2, light yellow and light green in Figure S10) along the resultant trisymmetron interface. The Glue protein components, consisting of 10 units, are located on the edge of the trisymmetron and serve to connect the adjacent trisymmetron. The bisymmetric structure looks like a dimer of the same protein, but it is unclear at this resolution. The Zipper protein components (orange in Figure 2, light green in Figure S10) bind to each of the two-fold symmetric Glue protein components (pink in Figure 2, light yellow in Figure S10). However, the two units near the Scaffold head (bracket in Figure 2 described in the next paragraph) are not well resolved (arrowheads in Figure 2).

These are internally supported by other protein components; one is the Scaffold protein component, and the other is the Support protein component (Figure 2). The Scaffold protein components (light yellow in Figure 2) show a paired head (bracket in Figure 2) and a following tailed structure, which are located anti-parallelly between pentasymmetrons along the trisymmetron interface. The Support protein components (dark blue in Figure 2) showed a large density and three additional smaller densities, running parallel to the trisymmetron interface and the Cement protein component array in each trapezoidal array. The three additional small densities were not observed in the tokyovirus capsid at 7.7 Å resolution [15] and only appear near the two-fold symmetry axis generated by the block-based reconstruction of the two-fold axis. As the Scaffold and Support protein components (light yellow and dark blue, respectively, in Figure 2) are underneath the lattice layer of the capsid consisting of MCP and mCPs, they are more flexible and will reduce resolution when the map is filtered by local resolution; however, without local filtering, they are exceedingly difficult to see (Figure 2).

### 3.6. Orientation of MCP Trimers on the Capsid Surface

**Figure 5:**
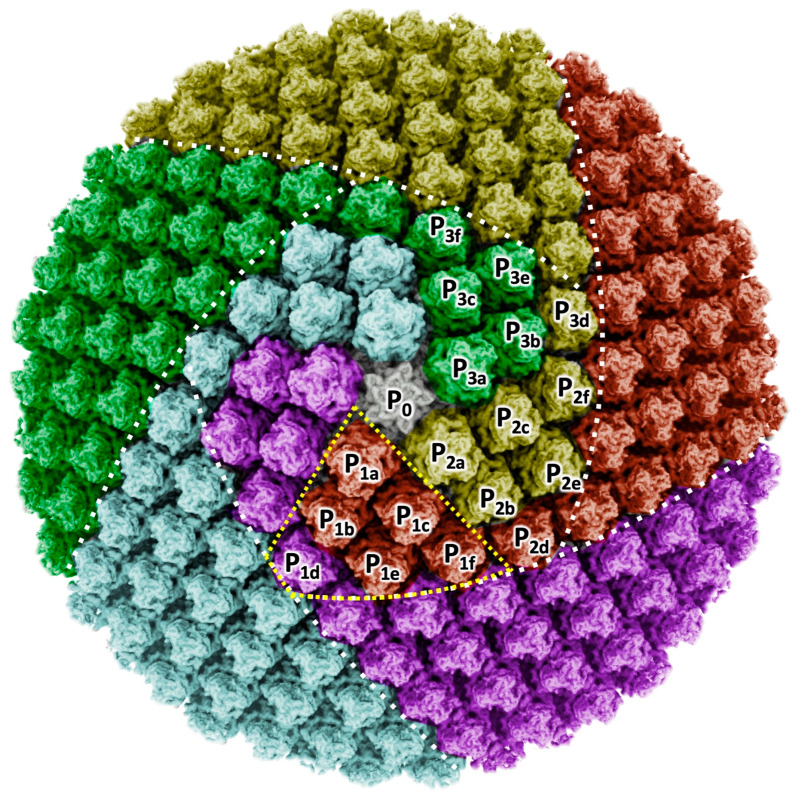
One of the pentasymmetron blocks, with MCPs colored by the orientations. The MCPs of melbournevirus show the same orientation pattern as CroV and PVCB-1 [16,26]. The white dotted lines show the interfaces of trisymmetron and pentasymmetron. The yellow dotted trapezoid shows an asymmetric unit of the pentasymmetron. Three asymmetric units of the pentasymmetron are labeled P1_a–f_, P2_a–f_, and P3_a–f_, showing that one of the MCPs in the asymmetric unit (P_1–3d_) is rotated 60° to match that of the adjacent asymmetric unit.

The pentasymmetron assembly of melbournevirus shares the MCP orientation (“golf club”) motif displayed in CroV [26] and PBCV-1 [16], in addition to that of the trisymmetron (Figure 5). In the motif, the corners, P*_nd_* (*n* = 1,2,3,4,5), of the MCP trimer in the asymmetric unit show the same 60° rotation as the adjacent asymmetric unit, which are shown with the same color in Figure 5. This is curious as Xian et al. hypothesize this orientational variance in the pentasymmetron asymmetric unit to be caused by the tape measure protein [16]. In melbournevirus, we have not found the clear density of the tape measure protein in the capsid, but instead a robust Scaffold array is localized between the pentasymmetrons (Figure 2), which were originally reported by Chihara et al. [15]. Examining under this “golf club” motif, PC-α and PC-β are placed at the interfaces of the individual motifs, and PC-γ is located at the interface of the trisymmetron motif adjacent to this motif (white circles in Figure 5). Looking at the minor capsid protein network in the melbournevirus capsid, the function of the tape measure proteins may be replaced by the coordinated use of PC-β and Scaffold proteins (Figure 2).

## 4. Discussion

This melbournevirus cryo-EM 3D reconstruction has a comparatively low particle count of 3124 particles when compared to the previously reported structures of PBCV-1 (~13,000 for a 3.5 Å reconstruction [17]) and ASFV (16,266 for a 14.1 Å reconstruction [18], 63,348 for an 8.8 Å reconstruction [19]). We attribute this to both biological and computational reasons. ASFV possesses an outer membrane, which will negatively impact the resolutions reported for a whole-virus reconstruction due to its disordered nature, which neither PBCV-1 nor melbournevirus possesses. PBCV-1, on the other hand, achieves a higher whole-virion resolution, which can be considered consistent with the increased particle count. It is also ~25% smaller than melbournevirus, at 190 nm, so the intra-particle defocus gradient should not be as large. Computationally, improvements in image processing software, specifically magnification anisotropy estimation and Ewald sphere correction, played a significant role in improving our melbournevirus reconstruction (see Section 2). As these computational methods advance further, we look forward to seeing further improvements for the study of giant viruses.

The melbournevirus MCP is structurally analogous to the PBCV-1 [17], Faustovirus [41], and ASFV [18,19] MCPs, with the sequence similarity including a double jelly roll motif, each composed of eight β-strands [42]. The greatest level of similarity is with tokyovirus (Figure S7) [15], where only a few residues differ, which would suggest the MCP structure is highly preserved in *Marseilleviridae*. With the improved resolution of the melbournevirus block reconstructions, we could better visualize the HI1 loop of melbournevirus MCP, contacting the HI2 loop of the adjacent monomer and forming a tight trimeric complex of MCP (Figure 3D,E and Figure S8A). This differs from ASFV, where it is the FG1 loop that is extended [18], and from PBCV-1, where a short FG1 loop is in close proximity to the HI2 loop of the neighboring MCP [43]. Furthermore, in melbournevirus MCP, the HI1 loop and the other external loops of DE1 and HI2 extend externally (Figure 3B and Figure S8) and form a cup structure on the top of the MCP trimer to accommodate additional cap densities (orange in Figure 3C). PBCV-1 MCP has a highly glycosylated “cap” region, while ASFV and faustovirus have a large additional β-sheet rich domain (Figure S8B–D). Melbournevirus, however, has an ordered region that is still lower in resolution than the main body of the MCP trimer (Figures S3D and S4D), which does not correspond to any part of the MCP model (Figure 3B,C). This ordered structural motif would imply the presence of protein, rather than sugars, which tend to be highly mobile. However, the reduced clarity of the density compared to that of the MCP may be caused by several factors. First, the cap may be in multiple conformations across the capsid surface, resulting in loss of clarity due to icosahedral averaging. Second, the cap may present partial occupancy of each MCP trimer across the capsid surface, also resulting in loss of clarity due to icosahedral averaging. To conclude this, further investigation is necessary to identify the cap protein and determine the stoichiometry with the MCP. As Fabre et al. [9], Okamoto et al. [14], and Jeudy et al. [39] report MEL_236 at equal or greater stoichiometry to the MCP protein, we can strongly assume that MEL_236 corresponds to the 14 kDa glycosylated cap protein we previously reported [15]. Aoki et al. reported that in hokutovirus and kashiwazakivirus, which belong to lineage B of *Marseilleviridae*, the newly replicated viruses are aligned on the surface of the host cells, exhibiting a “bunch” formation consisting of normal and infected cells, similar to tupanvirus [8]. The flexible characteristics of this glycosylated cap protein on the MCP trimer may be different within the members of *Marseilleviridae*, playing a different functional role in the interface against the viral host.

With the improvement in resolution of this melbournevirus reconstruction, we have been able to build a polyalanine model of the Penton base protein (Figure 4). The DeepTracer (online at: https://deeptracer.uw.edu, accessed on 31 March 2026) [35] chain tracing predicted an SJR fold (Figure S9). When comparing the DeepTracer-predicted structure to that of the PBCV-1 Penton base protein [17], the SJR fold aligns, but the less structured loops are of varying lengths. The polyalanine model fits the density well; however, the loops show some dislocation from that of PBCV-1 (Figure S9), which may explain why we have been unable to identify candidate Penton protein primary sequence via BLAST 2.10.0 [44,45] sequence homology. The CroV-dependent mavirus Penton [46], which we previously tested against tokyovirus, again aligns at the SJR fold but is otherwise a poor fit, even when the external insertion domain (curly bracket in Figure S9C), which is absent in PBCV-1 and Marseilleviruses, is ignored. The Penton shows more density underneath the fitted polyalanine model (arrow in Figure 4B). Weng et al. report that the Penton base pentamer hung lantern-like densities underneath the protein, connecting the Penton and internal membrane [19]. In the melbournevirus Penton, we do not find the lantern-like density under the Penton. The low-resolution density following the Penton (arrow in Figure 1B) may have a similar function to connect the Penton with the internal membrane extrusion.

There are two orientation combinations between MCP trimers, a non-rotating combination and a 60-rotating combination [19]. In the non-rotating combination, a jelly roll motif 1 (JR1) in the MCP trimer interacts with a jelly roll motif 2 (JR2) in the adjacent MCP trimer, creating a relatively flat MCP array, like the trisymmetron surface. In contrast, in the 60-rotating combination, a JR1 in the MCP trimer interacts with a JR1 in the adjacent MCP trimer, while a JR2 in the MCP trimer interacts with a JR2 in the adjacent MCP trimer. These combinations create a relatively angled edge, like the trisymmetron interface. In the pentasymmetron, the orientation of the MCP trimers is more complicated. Five asymmetric units of the pentasymmetron, ideally each consisting of six non-rotating combination MCP trimers, surround the central Penton protein, and the interface of each asymmetric unit interacts with the 60-rotating MCP trimer of the neighboring asymmetric unit. Finally, these MCP trimers create a Penton vertex (Figure 5), where Xiao et al. claimed in PBCV-1 and CroV that one of the six MCP trimers of the asymmetric unit, which is located at the corner of the pentasymmetron, was exceptionally rotated 60° [16,26]. They called this MCP trimer array of the asymmetric unit the “golf club motif”. Furthermore, the “golf club motif” was hypothesized to be caused by a specific localization of the tape measure proteins [16]. In our observations, melbournevirus showed a “golf club motif” of the MCP trimer array in the same orientation as PBCV-1 and CroV (Figure 5), where the rotation of the single MCP trimer by 60° (P_1d_, P_2d_, and P_3d_ in Figure 5) orients it to match that of the adjacent trisymmetron and Penton asymmetric unit. If we picture all the MCP trimers in a pentasymmetron asymmetric unit in the same orientation (Figure S11), we can better visualize why the rotation of a single MCP trimer is necessary. The single MCP trimer would cause a mismatch in the MCP orientational alignment of the trisymmetron interface (yellow arrows in Figure S11). The trisymmetron interface demonstrates a greater angle across it than the trisymmetron, which is of a gentler curvature. As such, it is likely that this orientational arrangement is a result of improving the flexibility of the capsid across the trisymmetron interface.

The Lattice proteins show strong similarity to the motifs displayed in the lower-resolution tokyovirus reconstruction. By using local resolution filtering in RELION 3.1 [24], we were able to avoid losing clarity in the more rigid Lattice proteins, while simultaneously visualizing the more flexible Scaffold array (Figure 2). Interestingly, this permitted visualization of three additional weak densities extending from the previously reported Support protein (dark blue in Figure 2), which run parallel to the Zipper/Glue proteins and Cement proteins on the trapezoidal lattice, but which themselves were not previously observed in tokyovirus. Missing them in tokyovirus was likely due to resolution limitations and caused by decreased signal-to-noise from fewer particles and the lack of dose weighting for cryo-HVEM data [15]. These may act to further support the lattice array as the array extends from the pentasymmetron construct along the trisymmetron interface, in addition to supporting the characteristic internal membrane structure.

Achieving close to the Nyquist limit of this dataset when reconstructing the complete viral particle with Ewald sphere correction demonstrates how vital proper analysis and correction of the various physical (electro-optical) effects can be for the study of giant viruses. The Ewald sphere reconstruction and solvent-corrected FSC curve do not fall to zero (black FSC curve, Figure S1J). Therefore, we also show the unmasked (non-solvent-corrected) Ewald-sphere-corrected curve (gray curve, Figure S1J) and the respective FSC curves without Ewald sphere correction (maroon and orange, respectively, Figure S1J), all of which fall to zero, indicating that the Ewald-sphere-corrected FSC curve not reaching zero is a result of how close it is to the maximum resolution. In our study, a processing method analogous to block-based reconstruction [20] (Figures S2–S5) was used to improve the clarity of the map further. When we used 1 MV cryo-HVEM for single-particle analysis (SPA) of tokyovirus [15], Ewald sphere correction did not improve the resolution beyond 7.7 Å. This suggests that the 300 kV microscope has a significant defocus gradient limitation for giant viruses above 200 nm, and thus, symmetry expansion and block-based reconstruction help counter this. Further, block-based reconstruction permits subtle variation in particle orientation, which is not possible with symmetry imposition. This may also play a role in improving resolution in the block reconstructions. However, as discussed in the previous paragraph regarding the weak density of cap proteins on MCPs, these averaging techniques may obscure potential capsid heterogeneity, in the same way that imposition of icosahedral symmetry would.

## 5. Conclusions

Here, we have increased the resolution of a cryo-EM 3D reconstruction of a Marseillevirus to a resolution approaching that necessary to build de novo structural models. This has allowed us to improve our previous marseillevirus MCP model and to begin the process of building a whole-capsid model. This is currently limited by both the lack of comprehensive annotation of capsid-related proteins and reaching the maximum resolution possible with this dataset. To fully clarify the structure of melbournevirus and build a complete model of the *Marseilleviridae* capsid, we must fully utilize our understanding of the genetics of the *Marseilleviridae*, using copy numbers to help identify the structural components and selected knockout genes to retrieve a phenotype, whilst simultaneously achieving resolutions that allow ab initio structure determination.

## Data Availability

The density maps have been deposited in the EMDB with the accession codes 31528 (whole virus), 31529 (five-fold block), 31530 (three-fold block), and 31531 (two-fold block). The atomic model of the MCP has been deposited in the PDB with the accession code 7YJL.

## Supplementary Materials

The following supporting information can be downloaded at https://www.mdpi.com/article/10.3390/v18040433/s1.
