# Supplementary material for: The 4.4 Å Capsid Structure of the Giant Melbournevirus Belonging to the Marseilleviridae Family
Source: Viruses. 2026 Apr 2;18(4):433. doi: 10.3390/v18040433 (PMC13119691; doi:10.3390/v18040433)
Supplement: Supplementary file 1 [file viruses-18-00433-s001.zip › viruses-4217449-supplementary.pdf]

Supplemental Information

The 4.4 Å capsid structure of the giant melbournevirus belonging to the *Marseilleviridae* family

Raymond N. Burton-Smith[1,2,3], Chantal Abergel[4], Kenta Okamoto[5], Kazuyoshi Murata[1,2,3*]

[1] Exploratory Research Center on Life and Living Systems (ExCELLS), National Institutes of Natural Sciences, Okazaki, Aichi, Japan

[2] National Institute for Physiological Sciences, National Institutes of Natural Sciences, Okazaki, Aichi, Japan

[3] Department of Physiological Sciences, School of Life Science, The Graduate University for Advanced Studies (SOKENDAI), Okazaki, Aichi, Japan

[4] Structural and Genomic Information Laboratory, UMR 7256 (IMM FR 3479, IM2B, IOM), Centre National de la Recherche Scientifique & Aix-Marseille University, Marseille 13288, France

[5] Program in Molecular Biophysics, Department of Cell and Molecular Biology, Uppsala University, Uppsala, Sweden

* Correspondence author and Lead contact: Kazuyoshi Murata (kazum@nips.ac.jp)

• Table S1-S2

• Figure S1 -S11

• References

Table S1. Dataset collection details

Melbournevirus data collection	Dataset 1	Dataset 2
Microscope	FEI Titan Krios G2	
Accelerating voltage (kV)	300	
Spherical aberration (mm)	2.7	
Detector	Gatan K2 Summit	
Total dose (e-/A^2)	26.4	
Micrographs	1,240	2,127
Frames per micrograph	20	40
Nominal magnification	64,000×	
Pixel spacing on the specimen (Å/pixel)	2.21	
Target defocus range (µm)	-0.8 – -3.0	
Initial particles picked	7,000	
Final particles used	3,124	

Table S2. Reconstruction details

Reconstruction	Whole virus	Five-fold block	Three-fold block	Two-fold block
Particles used	3,124	187,440	187,440	187,440
Symmetry imposed	I3	C1	C1	C1
Resolution	4.9	4.42	4.42	4.43
EMDB accession code	31528	31529	31530	31531

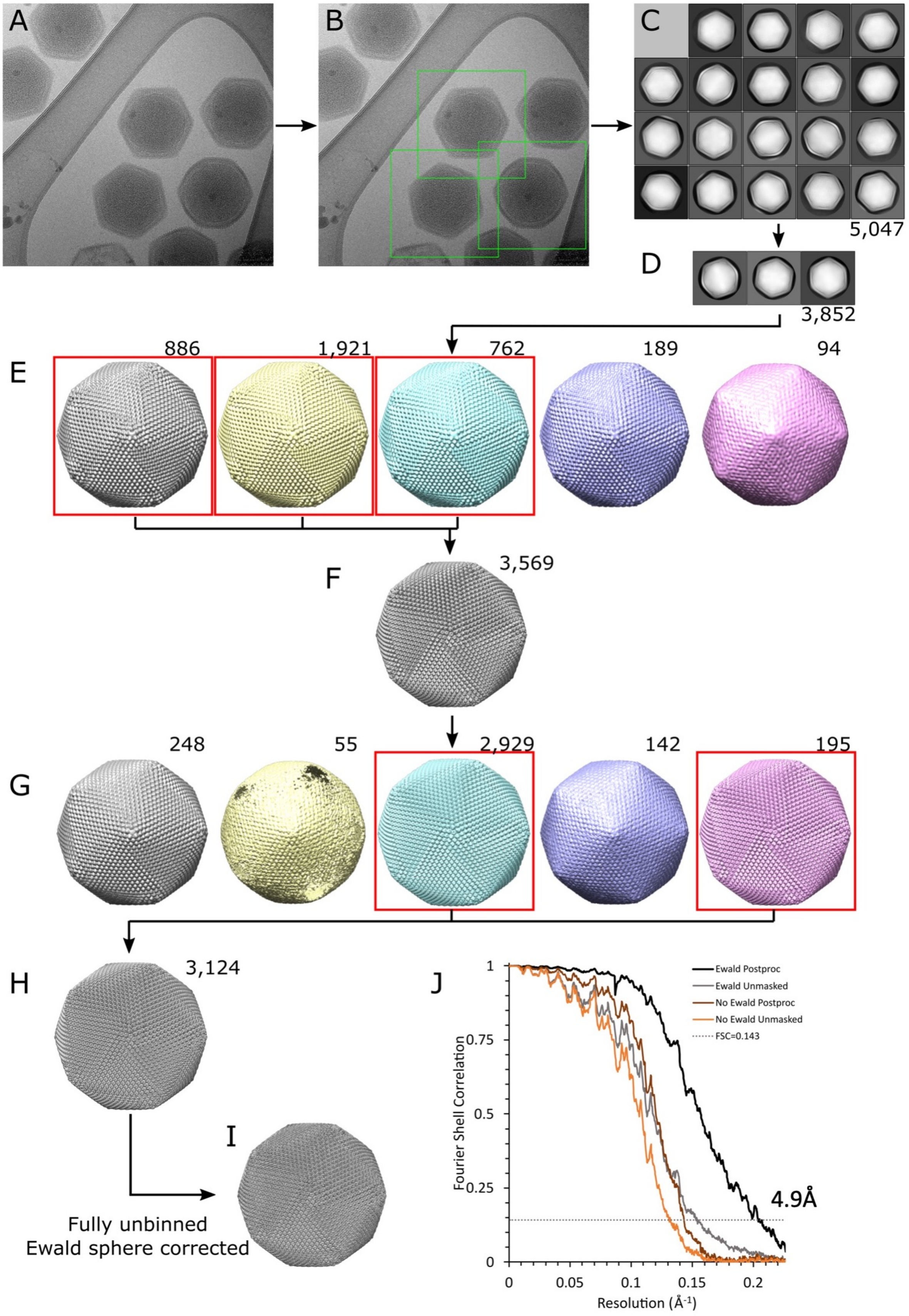
A
B
C
5,047
D
3,852
E
886
1,921
762
189
94
F
3,569
G
248
55
2,929
142
195
H
3,124
I
Fully unbinned
Ewald sphere corrected
J
Ewald Postproc
Ewald Unmasked
No Ewald Postproc
No Ewald Unmasked
FSC=0.143
Fourier Shell Correlation
Resolution (Å⁻¹)
4.9Å
0
0.05
0.1
0.15
0.2
0.25
0.5
0.75
1

Figure S1. Processing flowchart for the melbournevirus whole-virus reconstruction. All processing was carried out in RELION [1–4]. See methods for a full description of processing. A) Representative micrograph. B) Representative micrograph with selected particles indicated with a green box. A total of 7,000 particles were autopicked. C) Selected 2D classes from the first round of 2D classification. The images are extracted with 4× downsampling. D) Selected 2D classes from the second round of 2D classification. E) The initial 3D classification into five classes, with selected classes boxed in red. F) The first 3D reconstruction from the selected particles. G) A final 3D classification, selected classes boxed in red. H) 3D refinement before fully unbinning particles. I) Ewald sphere corrected final reconstruction. J) Global gold-standard Fourier shell correlation curves of the final reconstruction masked and unmasked, with and without Ewald sphere correction. Numbers by (C, D) indicate total selected particles. Numbers by 3D models (E–H) indicate number of particles within that class.

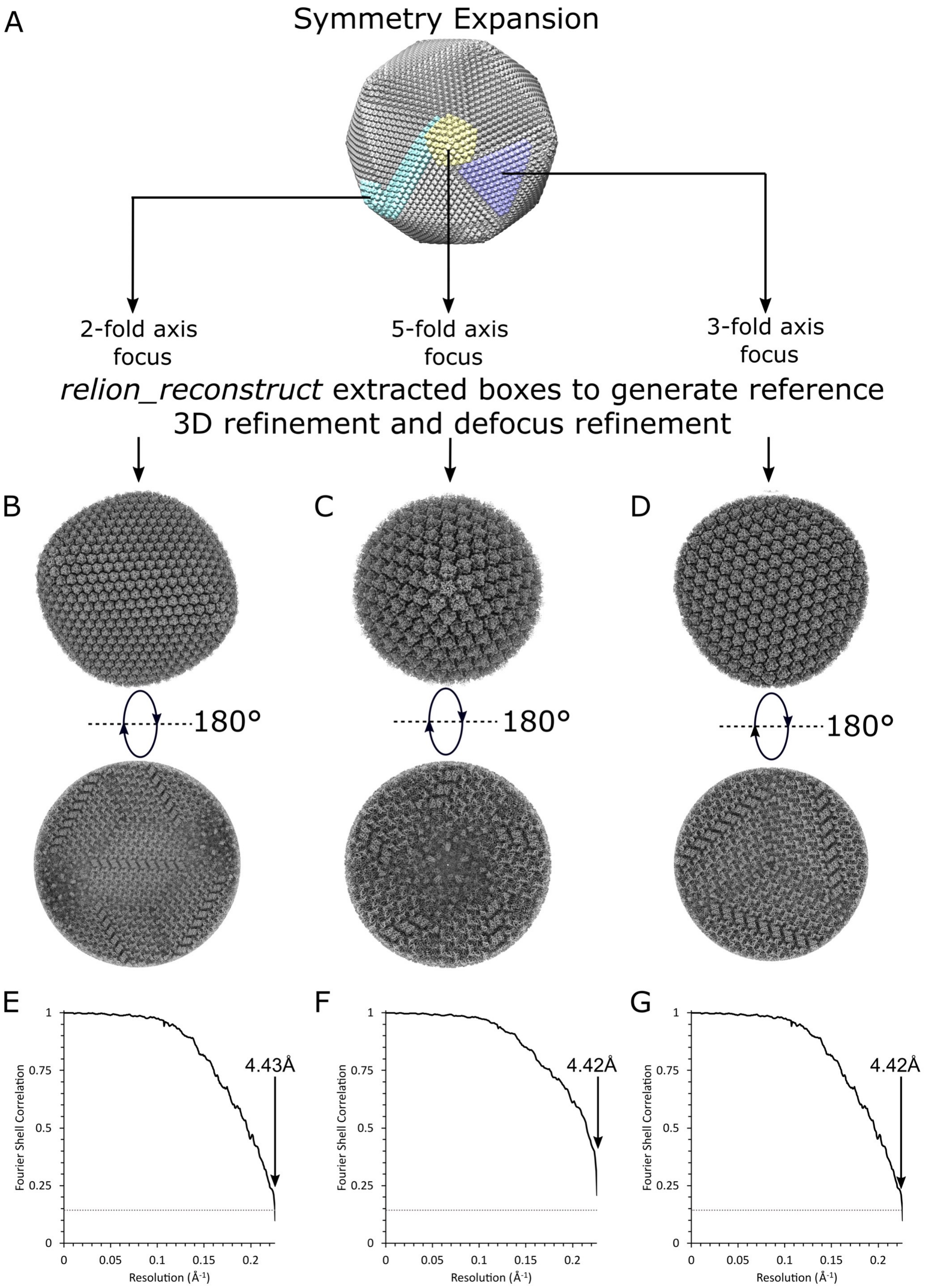
A
Symmetry Expansion
2-fold axis focus
5-fold axis focus
3-fold axis focus
relion_reconstruct extracted boxes to generate reference
3D refinement and defocus refinement
B
C
D
180°
180°
180°
E
F
G
4.43Å
4.42Å
4.42Å
Fourier Shell Correlation
Resolution (Å-1)

Figure S2. Processing flowchart of the melbournevirus block-based reconstructions. See methods for a full description of processing. A) The 4.9 Å whole-virus reconstruction, with the five-fold, three-fold and two-fold axes masked and coloured in yellow, purple, and cyan, respectively. Symmetry expansion was carried out resulting in 187,440 particles for each block, and the 3D refinements of the blocks were carried out. A single pass of defocus refinement was used before a final reconstruction. B) two-fold axis 3D reconstruction, external and internal views. C) Five-fold axis 3D reconstruction, external and internal views. D) Three-fold axis 3D reconstruction, external and internal views. E, F, G) Gold-standard Fourier shell correlation curves for the two-fold axis block, the five-fold axis block, and the three-fold axis block, respectively. All defocus-refined block reconstructions achieve Nyquist frequency.

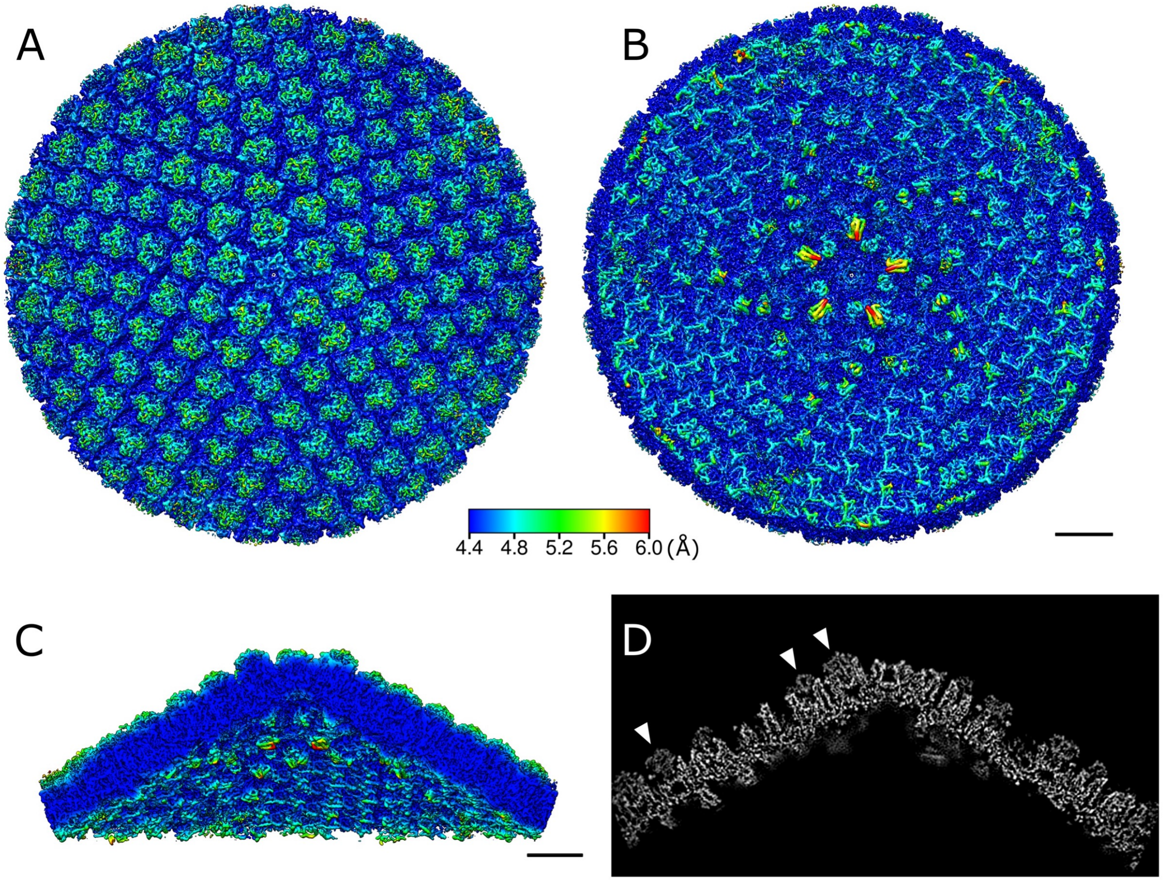

Figure S3. Block-based reconstruction of a five-fold axis point. A) External view. B) Internal view. C) Slice view perpendicular to (A) and (B). The map is colour coded according to the local resolution of the area. The colour code is shown in the figure. D) Slice view of density. White arrowheads indicate a centrally sliced MCP, showing the “cup” in the MCP which is filled with a cap density (arrowheads). Scale bars equal 10 nm.

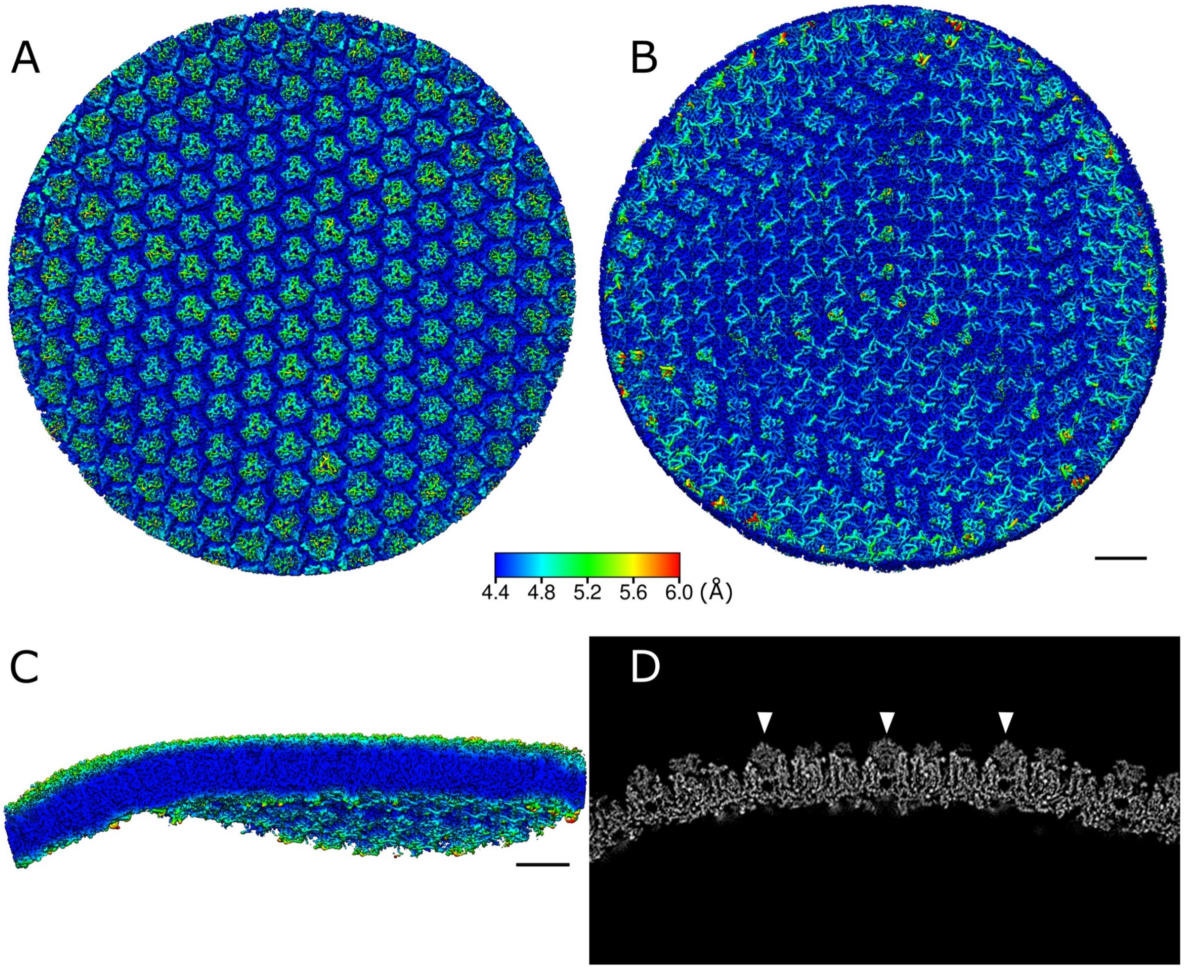

Figure S4. Block-based reconstruction of a three-fold axis point. A) External view. B) Internal view. C) Slice view perpendicular to (A) and (B). The map is colour coded according to the local resolution of the area. The colour code is shown in the figure. D) Slice view of density map. White arrowheads indicate a centrally sliced MCP, showing strong density for main MCP body and weaker density for the cap region (arrowheads), which fills the “cup” of the MCP. Scale bars equal 10 nm.

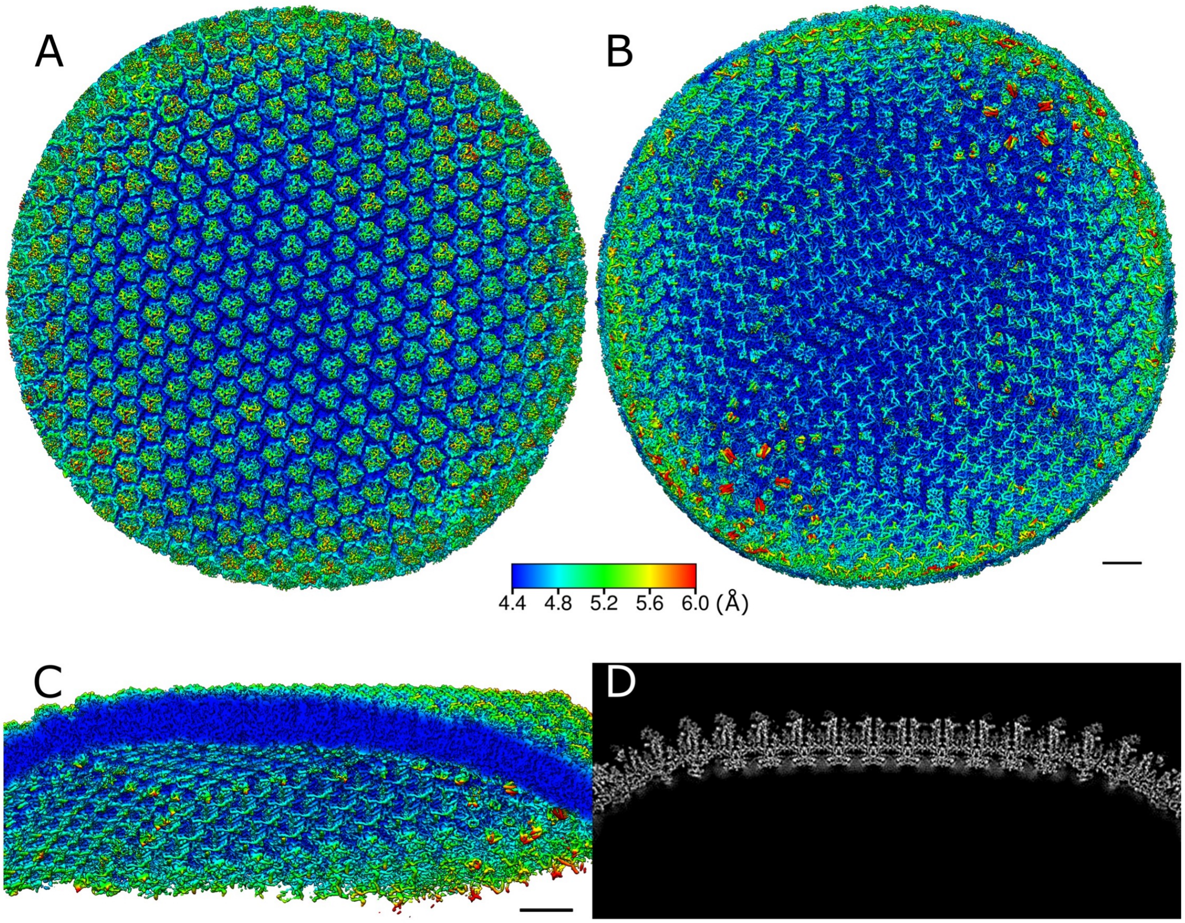

Figure S5. Block-based reconstruction of a two-fold axis point. A) External view. B) Internal view. C) Slice view perpendicular to (A) and (B). The map is colour coded according to the local resolution of the area. The colour code is shown in the figure. D) Slice view of the density map. Scale bars equal 10 nm.

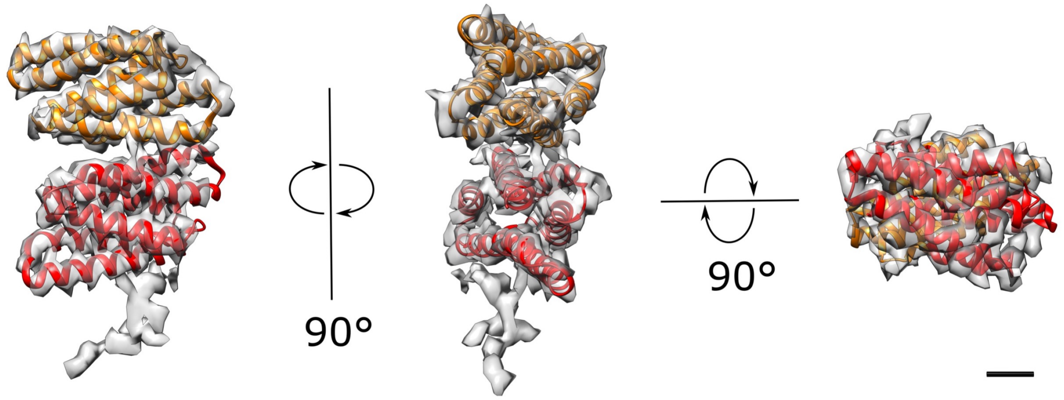

Figure S6. PC-α of melbournevirus, with a *de novo* fitted polyalanine model. The mCP consists of two quadruple bundles of α-helices, stacked one on top of the other. Scale bar equals 1 nm.

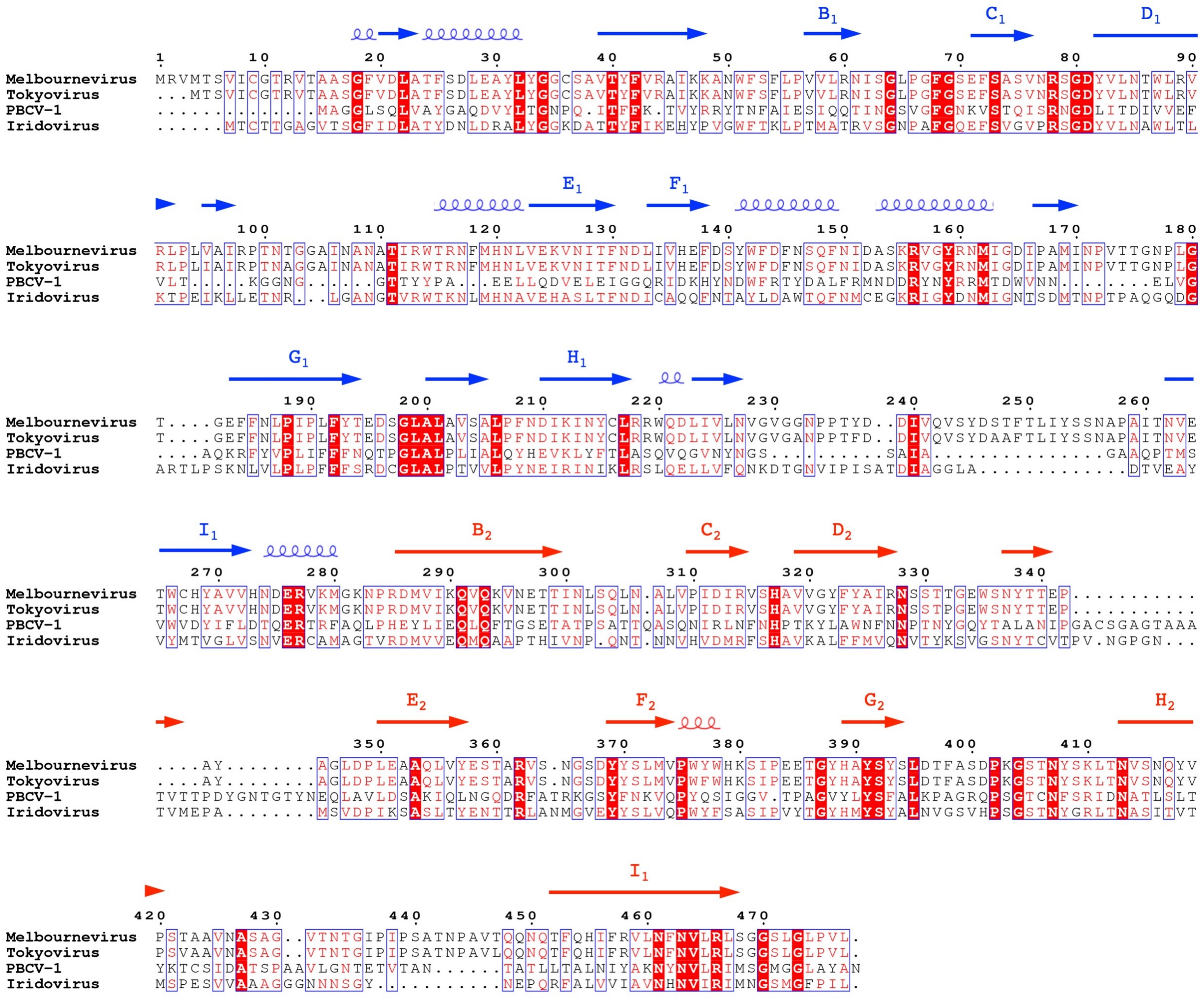

Figure S7. An alignment of melbournevirus MCP against three other NCLDV MCPs [5–7] , highlighting secondary structures: it is a helix with a coil and a β-sheet with an arrow. PROMALS3D [8] and ESpript3 [9] were used. Labelled blue and red arrows indicate areas of β-sheet comprising the first and second jelly roll fold, respectively.

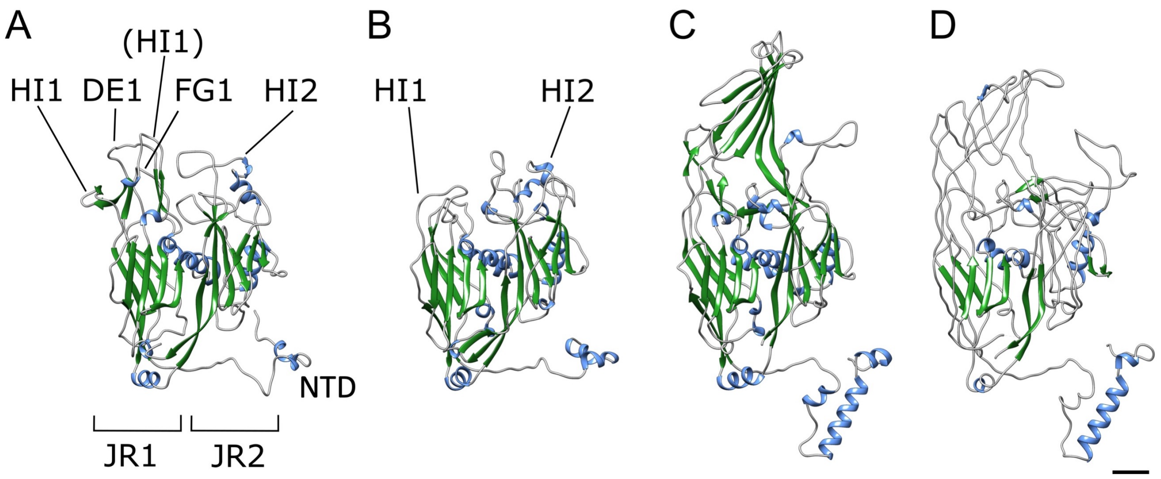

Figure S8. Comparison of the MCP monomers of melbournevirus (A), PBCV-1 (B) [6], faustovirus (C) [10], and ASFV (D) [11]. The secondary structures are coloured as follows: Random coil (grey), α-helix (blue) and β-sheet (green). The names of individual loops are indicated. JR1 and JR2 show jelly roll motif 1 and 2, respectively. NTD represents the N-terminal domain. Scale bar equals 1 nm.

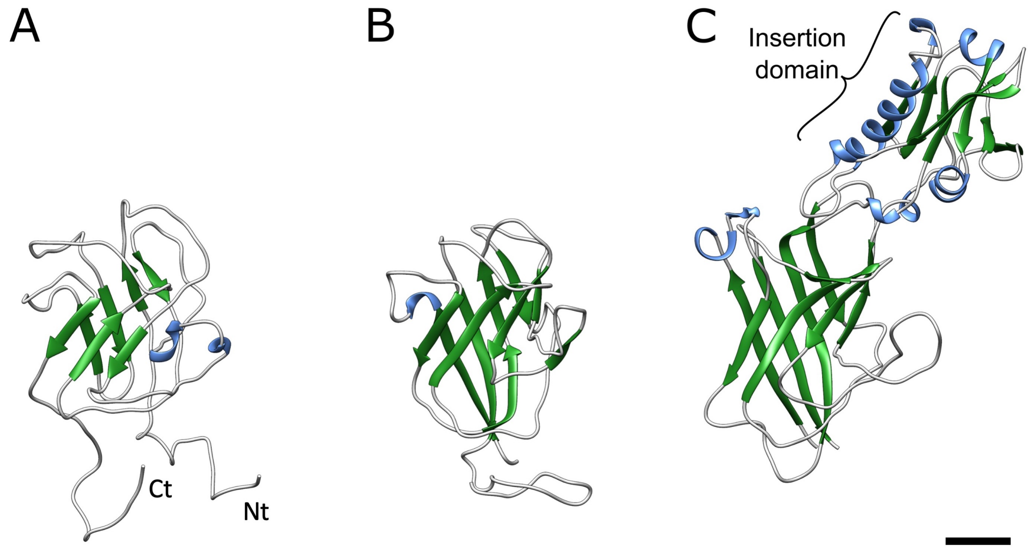

Figure S9. The ribbon models of the Penton based protein of melbournevirus (A), and PBCV-1 (B) [6], and CroV-dependent mavirus (C) [12] were compared using a single jellyroll motif. Ct and Nt show the C-terminal and N-terminal, respectively. Scale bar equals 1 nm.

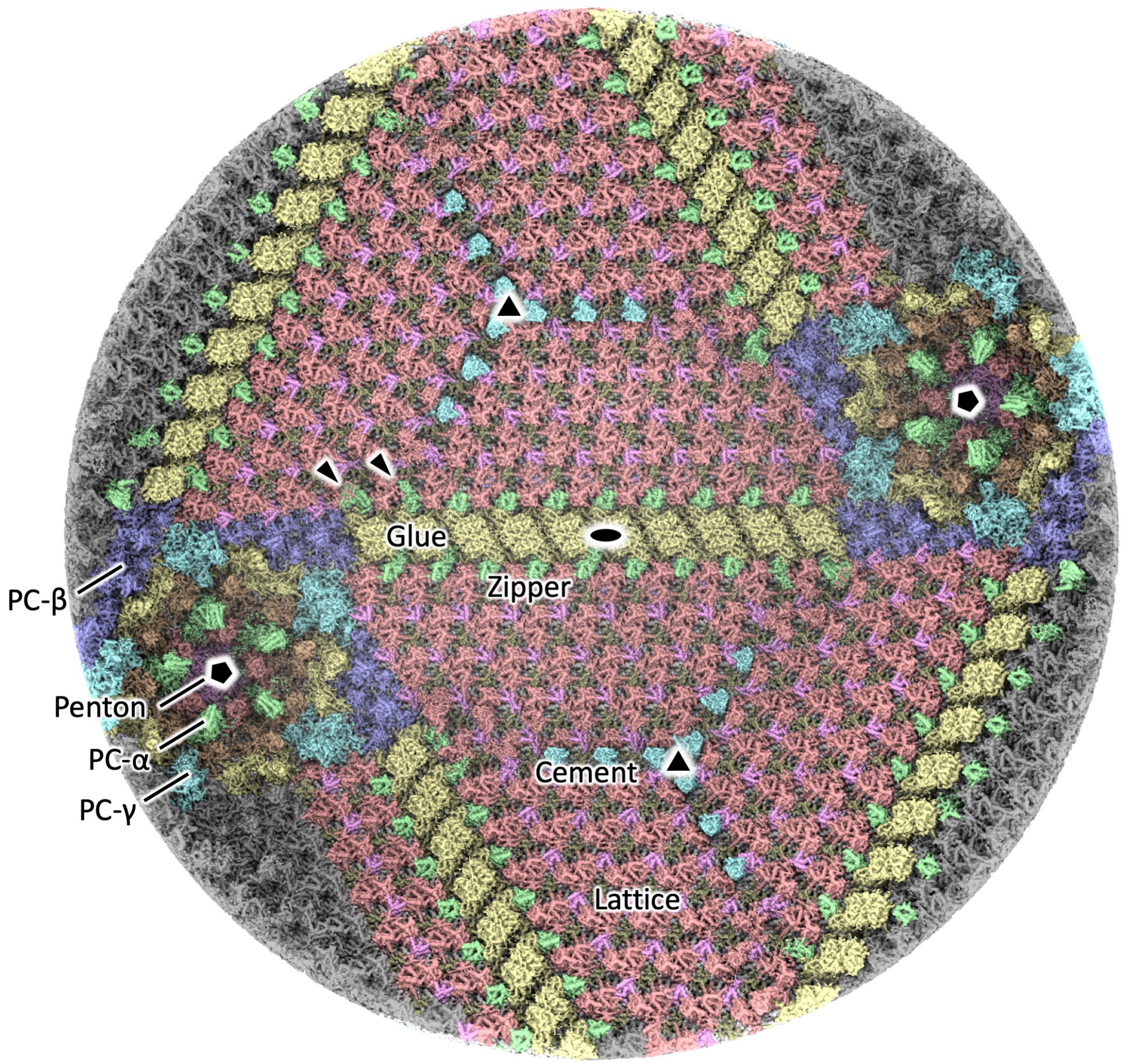

Figure S10. Segmentation of the mCPs of melbournevirus. The Scaffold and Support protein components located between the mCP and the internal membrane have been removed from Fig. 2, allowing for clear identification of the mCP components.

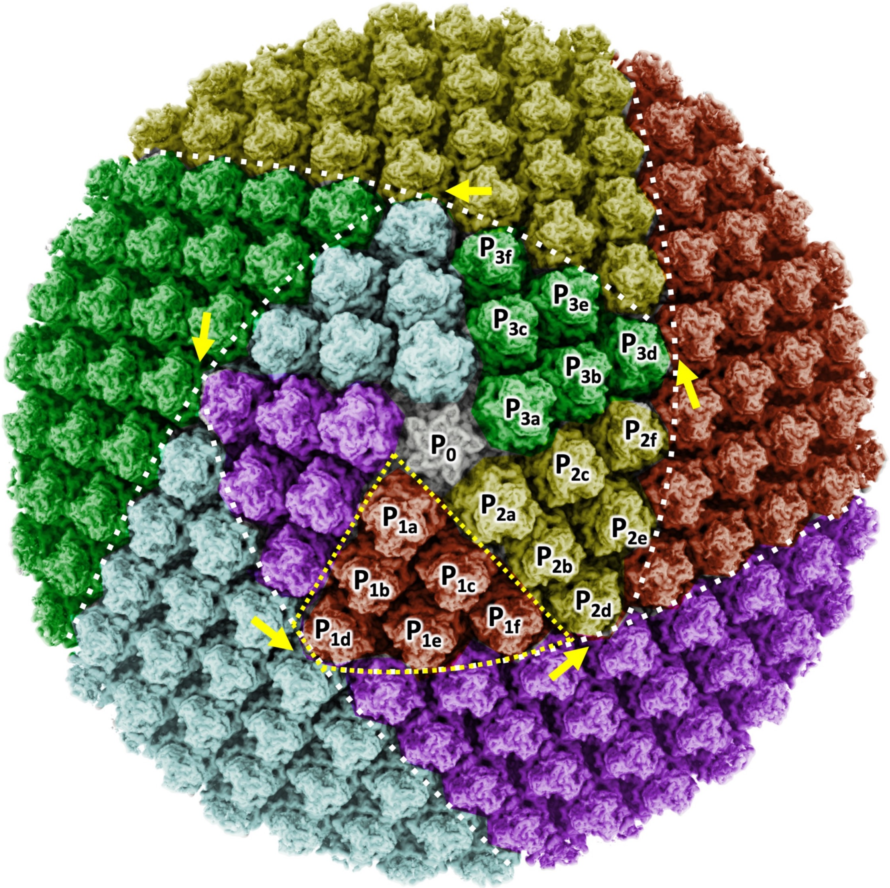

Figure S11. Alternate colouring of Fig. 5, if all the MCP trimers of the pentasymmetron asymmetric unit were oriented the same. The white dotted lines indicate the interfaces of trisymmetron and pentasymmetron. The yellow dotted trapezoid shows an asymmetric unit of the pentasymmetron. The MCPs in the three asymmetric units of the pentasymmetron are labelled $P_{1a\text{-}f}$, $P_{2a\text{-}f}$ and $P_{3a\text{-}f}$, indicating that one of the MCPs in the asymmetric unit ($P_{1d\text{-}3d}$) is not rotated by 60°. Resultantly, the single MCP trimer would cause a mismatch in the MCP orientational alignment of the trisymmetron interface (yellow arrows). This shows how the smooth interface along the edge of the trisymmetron would be broken, potentially disrupting capsid formation.

Supplementary References

1. Fernandez-Leiro, R.; Scheres, S.H.W. A Pipeline Approach to Single-Particle Processing in RELION. *Acta Crystallogr. D Struct. Biol.* **2017**, *73*, 496–502, doi:10.1107/S2059798316019276.
2. Scheres, S.H.W. RELION: Implementation of a Bayesian Approach to Cryo-EM Structure Determination. *J. Struct. Biol.* **2012**, *180*, 519–530, doi:10.1016/j.jsb.2012.09.006.
3. Zivanov, J.; Nakane, T.; Forsberg, B.; Kimanius, D.; Hagen, W.J.H.; Lindahl, E.; Scheres, S.H.W. New Tools for Automated High-Resolution Cryo-EM Structure Determination in RELION-3. *Elife* **2018**, *7*, e42166, doi:10.1101/421123.
4. Zivanov, J.; Nakane, T.; Scheres, S.H.W. Estimation of High-Order Aberrations and Anisotropic Magnification from Cryo-EM Data Sets in RELION-3.1. *IUCrJ* **2020**, *7*, 253–267, doi:10.1107/S2052252520000081.
5. Chihara, A.; Burton-Smith, R.N.; Kajimura, N.; Mitsuoka, K.; Okamoto, K.; Song, C.; Murata, K. A Novel Capsid Protein Network Allows the Characteristic Internal Membrane Structure of Marseilleviridae Giant Viruses. *Scientific Reports 2022 12:1* **2022**, *12*, 1–15, doi:10.1038/s41598-022-24651-2.
6. Fang, Q.; Zhu, D.; Agarkova, I.; Adhikari, J.; Klose, T.; Liu, Y.; Chen, Z.; Sun, Y.; Gross, M.L.; Van Etten, J.L.; et al. Near-Atomic Structure of a Giant Virus. *Nat. Commun.* **2019**, *10*, 388, doi:10.1038/s41467-019-08319-6.
7. Pintilie, G.; Chen, D.H.; Tran, B.N.; Jakana, J.; Wu, J.; Hew, C.L.; Chiu, W. Segmentation and Comparative Modeling in an 8.6-Å Cryo-EM Map of the Singapore Grouper Iridovirus. *Structure* **2019**, *27*, 1561-1569.e4, doi:10.1016/j.str.2019.08.002.

8. Pei, J.; Kim, B.H.; Grishin, N. V. PROMALS3D: A Tool for Multiple Protein Sequence and Structure Alignments. *Nucleic Acids Res.* **2008**, *36*, 2295–2300, doi:10.1093/nar/gkn072.

9. Gouet, P.; Robert, X.; Courcelle, E. ESPript/ENDscript: Extracting and Rendering Sequence and 3D Information from Atomic Structures of Proteins. *Nucleic Acids Res.* **2003**, *31*, 3320–3323, doi:10.1093/NAR/GKG556.

10. Klose, T.; Reteno, D.G.; Benamar, S.; Hollerbach, A.; Colson, P.; La Scola, B.; Rossmann, M.G. Structure of Faustovirus, a Large DsDNA Virus. *Proceedings of the National Academy of Sciences U. S. A.* **2016**, *113*, 6206–6211, doi:10.1073/pnas.1523999113.

11. Wang, N.; Zhao, D.; Wang, J.; Zhang, Y.; Wang, M.; Gao, Y.; Li, F.; Wang, J.; Bu, Z.; Rao, Z.; et al. Architecture of African Swine Fever Virus and Implications for Viral Assembly. *Science (1979).* **2019**, *366*, 640–644, doi:10.1126/science.aaz1439.

12. Born, D.; Reuter, L.; Mersdorf, U.; Mueller, M.; Fischer, M.G.; Meinhart, A.; Reinstein, J. Capsid Protein Structure, Self-Assembly, and Processing Reveal Morphogenesis of the Marine Virophage Mavirus. *Proc. Natl. Acad. Sci. U. S. A.* **2018**, *115*, 7332–7337, doi:10.1073/pnas.1805376115.
